# The Variants at *APOA1 a*nd *APOA4* Contribute to the Susceptibility of Schizophrenia With Inhibiting mRNA Expression in Peripheral Blood Leukocytes

**DOI:** 10.3389/fmolb.2021.785445

**Published:** 2021-12-06

**Authors:** Yao Fan, Jun Gao, Yinghui Li, Xuefei Chen, Ting Zhang, Weiyan You, Yong Xue, Chong Shen

**Affiliations:** ^1^ Department of Clinical Epidemiology, Jiangsu Province Geriatric Institute, Geriatric Hospital of Nanjing Medical University, Nanjing, China; ^2^ Department of Epidemiology, School of Public Health, Nanjing Medical University, Nanjing, China; ^3^ Department of Neurobiology, Nanjing Medical University, Nanjing, China; ^4^ Department of Medical Psychology, Huai’an Third Hospital, Huai’an, China; ^5^ Department of Medical Laboratory, Huai’an Third Hospital, Huai’an, China

**Keywords:** schizophrenia, APOA1 gene, APOA4 gene, mRNA, case–control study, apoA1, HDLC

## Abstract

**Objective:** Abnormal lipid metabolism has a close link to the pathophysiology of schizophrenia (SZ). This study mainly aimed to evaluate the association of variants at apolipoprotein A1 (*APOA1*) and *APOA4* with SZ in a Chinese Han population.

**Methods:** The rs5072 of *APOA1* and rs1268354 of *APOA4* were examined in a case–control study involving 2,680 patients with SZ from the hospital and 2,223 healthy controls screened by physical examination from the community population. The association was estimated with the odds ratio (OR) and 95% confidence intervals (95% CIs) by logistic regression. The *APOA1* and *APOA4* messenger RNA (mRNA) in peripheral blood leukocytes were measured by real-time PCR and compared between SZ cases and controls. Serum apoA1 levels were detected by turbidimetric inhibition immunoassay and high-density lipoprotein cholesterol (HDL-C) levels were detected by the homogeneous method.

**Results:** Both of the rs5072 of *APOA1* and rs1268354 of *APOA4* had statistically significant associations with SZ. After adjustment for age and sex, ORs (95% CIs) of the additive model of rs5072 and rs1268354 were 0.82 (0.75–0.90) and 1.120 (1.03–1.23), and *p*-values were 3.22 × 10^−5^ and 0.011, respectively. The association of rs5072 with SZ still presented statistical significance even after Bonferroni correction (*p*-value×6). SZ patients during the episode presented lower levels of apoA1, HDL-C, mRNA of *APOA1* common variants and transcript variant 4, and *APOA4* mRNA than controls (*p* < 0.01) while SZ patients in remission showed a significantly decreased *APOA1* transcript variant 3 expression level and increased *APOA4* mRNA expression level (*p* < 0.01). mRNA expression levels of *APOA1* transcript variant 4 significantly increased with the variations of rs5072 in SZ during the episode (*p*_trend_ = 0.017). After the SZ patients received an average of 27.50 ± 9.90 days of antipsychotic treatment, the median (interquartile) of serum apoA1 in the SZ episode significantly increased from 1.03 (1.00.1.20) g/L to 1.08 (1.00.1.22) g/L with the *p*-value of 0.044.

**Conclusion:** Our findings suggest that the genetic variations of *APOA1* rs5072 and *APOA4* rs1268354 contribute to the susceptibility of SZ, and the expression levels of *APOA1* and *APOA4* mRNA of peripheral blood leukocytes decreased in SZ patients during the episode while *APOA4* increased after antipsychotic treatment.

## Introduction

Schizophrenia (SZ) is a severe chronic psychiatric disorder characterized by distorted thinking processes, hallucinations, delusions, and functional deterioration (Barnett, 2018; Charlson et al., 2019). Lifetime risk for developing SZ, in general, is estimated to be approximately 1% (McGrath et al., 2008; Huang et al., 2019). The global age-standardized point prevalence of schizophrenia in 2016 was estimated to be 0.28% (95% UI: 0.24–0.31) (Charlson et al., 2018). This disorder accounted for 7.4% (5.0%–9.8%) of the fifth leading disorder in the disability-adjusted life year (DALY) (Collins et al., 2011). People suffering from schizophrenia often experience a decline in the quality of life, are prone to suicide, have an increased risk of comorbidities, and have a high mortality rate (Rossler et al., 2005; Tan et al., 2021). Due to its early age of onset and permanent disability, SZ invariably brings emotional and financial devastation to its victims and their families. Thus, SZ is considered to be one of the most catastrophic mental illnesses (Ayalew et al., 2012). However, to date, there is still no effective method available for the prevention and treatment of SZ.

SZ is a complex psychosis caused by genetic and environmental factors (Perzel Mandell et al., 2021; Robinson and Bergen, 2021). Previous studies suggest that abnormal lipid metabolism is closely related to the dysfunction of monoamine neurotransmitters (Lewis et al., 2010) and may contribute to the pathophysiology of SZ (La et al., 2007; Keeney et al., 2013; Song et al., 2014). Low levels and activity of ApoA1 might be a cause and consequence of immune-inflammatory and oxidative stress pathways in SZ patients (Morris et al., 2021). ApoA1 is the major apolipoprotein of HDL-C, which plays a key role in the process of cholesterol reverse transport, phospholipid efflux, and lipoprotein metabolism(Boiko et al., 2019). Several studies have shown that the decreased level of plasma, serum, or cerebrospinal fluid (CSF) apoA1is associated with a variety of neurodegenerative diseases, such as Alzheimer’s disease (AD), Parkinson’s disease (PD), and SZ (Liu et al., 2006; Huertas et al., 2017).

ApoA1 is encoded by the *APOA1* gene (Saito et al., 2004). In the human body, the *APOA1* gene is localized at chromosome 11q23 (11q23.3), which has been a popular locus for SZ research (Mack et al., 2017). Some studies have suggested that the abnormalities of chromosome 11q23 increase the risk for SZ (Gurling et al., 2001; Demirhan and Tastemir, 2003; Lewis et al., 2003).

Apolipoprotein A4 (apoA4), a component of lipoprotein particles, has been suggested to play an important role in brain metabolism. In 1977, apoA4 was first described to be a component of chylomicrons and HDL-C in the rat (Swaney et al., 1977) and detected in astrocytes of the rat. In fasting plasma, apoA4 is mainly found in HDL-C (Green et al., 1980). *APOA4* is also localized at chromosome 11q23.3. In the meantime, it has been suggested that *APOA1* and *APOA4* genes, which are transcribed in the same direction, have an evolutionary relationship and come from the same ancestral sequence (Karathanasis, 1985).

Although genome-wide association studies (GWAS) of SZ have not identified susceptible loci linked to APOA1 and APOA4 yet, the aforementioned evidence suggests that APOA1 and APOA4 may be involved in SZ pathophysiology whereas the molecular mechanism is still unclear. Therefore, this study aimed to investigate the association of variants at *APOA1* and *APOA4* with SZ, compare *APOA1* and *APOA4* mRNA expression levels in peripheral blood leukocytes between SZ cases and controls, and evaluate the influence of *APOA1* and *APOA4* variations on the mRNA expression level*.*

## Methods

### Study Population

A total of 2,680 patients with SZ (1,229 men and 1,451 women) were consecutively recruited from the Third People’s Hospital of Huai’an, Jiangsu Province, China, from the year 2009–2018. All patients were diagnosed with SZ according to the Diagnostic and Statistical Manual fourth edition (DSM-IV), and were diagnosed by at least two experienced psychiatrists. A total of 2,223 healthy controls (including 1,365 men and 858 women) screened by the physical examination were recruited from the local community, and those who had a personal or family history of psychiatric disorders were excluded. All participants were free of cardiovascular diseases, cerebral vascular diseases, serious infections, and surgery. The controls had a higher average age [(43.71 ± 8.79) years] than SZ cases [(34.69 ± 13.00) years]. Thus, the controls would have less risk to develop into SZ, and that would help to enrich the chance to find susceptible genetic loci.

This study was done in full compliance with the Helsinki Declaration. The protocol and consent form were approved by the Institutional Review Board of Nanjing Medical University, China. All participants or their guardians provided signed informed consents.

### Data Collection

Questionnaire interviews were conducted to collect information on participants’ age, sex, nationality, history of chronic diseases, and family status.

Total cholesterol (TC), low-density lipoprotein cholesterol (LDL-C), apoA1, high-density lipoprotein cholesterol (HDL-C), triglycerides (TG), and glucose (GLU) were detected by Total Cholesterol Kit (CHOD-PAP Method), Low-Density Lipoprotein Cholesterol Kit (Clearance Method), Turbidimetric inhibition immunoassay, High-Density Lipoprotein Cholesterol Kit (Clearance Method), enzymatic method, and Glucose oxidase, respectively. Total bilirubin (TBIL) and direct bilirubin (DBIL) were examined by the vanadate oxidation method. Indirect bilirubin (IBIL) is the difference between total bilirubin (TBIL) and direct bilirubin (DBIL). All biochemistry indices were detected in the Third People’s Hospital of Huai’an.

### SNP Selection

*APOA1* [GenBank ID:335 also known as apo(a)] and *APOA4* (GeneBank ID:337), encoding apoA1 and apoA4 protein, respectively, were both mapped on chromosome 11p23.3. We searched for seven SNPs (rs5072, rs2070665, rs10750098, rs632153, rs12718462, and rs12718464 of *APOA1*, and rs1268354 of *APOA4*) covering the *APOA1* and *APOA4* from the upstream 2 kb to the downstream 1 kb, and further selected tag SNPs (tagSNPs) from the database of the Genome Variation Server 147 (GVS) (http://gvs.gs.washington.edu/GVS147/).

SNPs with minor allele frequency (MAF) > 0.05 will be included as candidate SNPs, and tagSNPs were selected for the criterion of linkage disequilibrium (LD) *r* ^2^ ≥ 0.8 in the GVS. Furthermore, the role of transcription, regulating, or splicing was evaluated on the website of selection tool for SNPs (SNPINFO, https://snpinfo.niehs.nih.gov/) and Regulome (http://www.regulomedb.org.). Thus, the SNPs with predicted biological functions of rs5072of *APOA1* (TF binding and DNase peak) and rs1268354 of *APOA4* (eQTL, TF binding and DNase peak) were preferentially included in this study. The biological information analysis for regions, alleles, MAFs, predicted functions, and Regulome DB score of the seven SNPs are shown in Supplementary Table S1. Primers and probes of rs5072 and rs1268354 are summarized in Supplementary Table S2.

### DNA Isolating and Genotyping

Genomic DNA was isolated from leukocytes of venous blood by proteinase K digestion and phenol-chloroform extraction. The primers and probes of TaqMan® assays were designed using Primer Express Oligo Design software v2.0 (ABI PRISM), and available upon request as TaqMan® Pre-Designed SNP Genotyping Assays. From randomly selected samples, 5% were genotyped again for quality control with complete concordance. PCR reactions and conditions are described at the Supplementary Information Genotyping PCR reactions and conditions.

### Determination of *APOA1* and *APOA4* mRNA in Peripheral Blood Leukocytes

A total of 61 patients with SZ during the acute episode lack systemic and effective anti-psychotic treatment, 64 patients with SZ in remission after a period of antipsychotic treatment and identified by clinicians, and 84 controls were selected to detect the expression level of *APOA1* and *APOA4* mRNAs. Fasting blood samples over 12 h of SZ patients on admission and on discharge were collected. Healthy controls were recruited from the local community population, and fasting blood samples over 12 h were collected. EDTA-containing blood sample was mixed with blood preservation solution (Eaglink Cat#EGEN2024, NANJING YININGFUSHENG Biotech. Co., Ltd. Nanjing, China) by 1:3. The total RNA was isolated from an 800-µl mixture using RNA Blood Kit (Cat#Yu-B02-1, Yuan Corp. Wuxi, China) according to the manual instructions. RNA quality and integrity were evaluated using electrophoresis on a 1.0% agarose gel and agarose gel and NanoDrop® ND ND-2000 spectrophotometer (NanoDrop, Wilmington, DE), respectively. cDNA was synthesized using TAKARA reverse transcription (RT) kits (RR047A Takara PrimeScript™ RT reagent Kit with gDNA Eraser, Japan). The quantitative real-time PCR (qPCR) reactions were performed in a 10-μl reaction mixture including 2 μl of cDNA and 5 μl of HieffTM qPCR SYBR® GEEN Master Mix. Primer sequences of *APOA1*, *APOA4*, and GAPDH (control) cDNAs are listed in Supplementary Table S3. The qPCR instrument used in this study was ABI RT-PCR 7900 (Applied Biosystems; Thermo Fisher Scientific, Inc.). Three parallel samples were set for each sample. The CT value results were read in the SDS2.4 software. The standard deviation of CT values between three parallel samples was less than 0.5, and finally we took the average number. Glyceraldehyde-3-phosphate dehydrogenase (GAPDH) was used as an internal control, and that could address blood sample-associated RNA quality issues in qPCR. The CT values among different plates had good and stable expressions. All the samples of SZ patients during the episode and in remission, and all control samples were arranged in one 384 plate to control variations. The relative expression of mRNA was calculated by 2^-∆∆CT^ (∆∆CT case = ∆CT case - ∆CT control average value, ∆∆CT control = ∆CT control - ∆CT control average value, ∆CT = CT target gene - CT housekeeper gene). All the reverse transcription and qPCR reactions, as well as conditions, are described at the Supplementary Information Reverse transcription reactions and conditions, and qPCR reactions and conditions.

### Statistical Analysis

Quantitative variables with normal distribution were expressed as mean ± standard deviation, and differences between cases and controls were assessed with unpaired Student’s *t*-test. ApoA1 concentration did not follow the normal distribution and thus was expressed as median (interquartile, IRQ). The differences of mRNA expression, apoA1, and HDL-C between SZ cases and controls, as well as among genotypes, were assessed with Mann–Whitney *U* test and Jonckheere–Terpstra test, respectively. Comparison of apoA1 and HDL-C levels before and after antipsychotic treatment was assessed by Wilcoxon test. Partial correlation between apoA1, HDL-C, and mRNA expression levels was estimated with adjustment for age and sex. Qualitative variables, the genotype, and allele frequency distributions between cases and controls were compared *via* the two-sided chi-square (*χ* ^2^) test. Among controls, genotype frequencies for each SNP were tested by Fisher’s exact *χ* ^2^ test using the program Hardy–Weinberg equilibrium (HWE). The association between loci and SZ was estimated by logistic regression. Odds ratios (OR) and 95% confidence intervals (CIs) were calculated. Then, we conducted a propensity matching analysis. We matched the SZ cases and controls by propensity score matching model of age and sex. Bonferroni correction was used for correcting the *p*-values for multiple comparisons. Statistical significance was set at two-tailed *p* < 0.05. All statistical analyses were performed with SPSS 18.0 (SPSS, Inc. Chicago, United States).

## Results

### Clinical Characteristics of Study Population

The general demographic characteristics of participants are summarized in Table 1. There were significant differences in age between SZ patients and controls (*t* = 27.86, *p* < 0.001). Sex differed significantly between the two groups (*χ* ^2^ = 117.85, *p* < 0.001). A total of 562 SZ patients (17.35%) had a family history of SZ and 2,118 patients (82.65%) did not. Among SZ patients, 116 (4.38%) patients were mainly treated with antipsychotics, 2,292 (86.52%) patients were treated with atypical antipsychotics, and 241 (9.10%) patients were treated with the combination of antipsychotics and atypical antipsychotics. All the biochemical indices were statistically lower in the cases than in the controls (*p* < 0.005).

**TABLE 1:** Demographic and clinical characteristics of SZ cases and controls.

Variable	Group	Case	Control	*t/χ* ^2^ */Z*	*p*
		(*n* = 2,680)	(*n* = 2,223)		
Age		34.69 ± 13.00	43.71 ± 8.79	27.86	<0.001
Sex	Men	1,229 (45.90%)	1,365 (61.40%)	117.85	<0.001
	Women	1,451 (54.10%)	858 (38.60%)		
Family history of SZ	Yes	562 (17.35%)			
	No	2,118 (82.65%)			
Subtypes of SZ	Paranoid SZ	572 (23.17%)			
	Amorphous SZ	1765 (71.49%)			
	Other subtypes	132 (5.34%)			
SZ drugs	Antipsychotics	116 (4.38%)			
	Atypical antipsychotics	2,292 (86.52%)			
	Combination therapy	241 (9.10%)			
GLU (mmol/L)		4.70 (4.20, 5.33)	5.45 (5.00, 5.94)	26.86	<0.001
TBIL (umol/L)		11.16 (8.12, 15.80)	11.90 (9.00, 16.00)	3.44	<0.001
DBIL (umol/L)		4.29 (2.94, 6.00)	5.00 (3.93, 6.20)	8.51	<0.001
IBIL (umol/L)		7.00 (4.93, 10.27)	7.74 (5.26, 11.34)	3.55	<0.001
TC (mmol/L)		3.96 (3.40, 4.62)	4.65 (4.06, 5.22)	22.24	<0.001
TG (mmol/L)		0.97 (0.69, 1.48)	1.29 (0.89, 2.00)	14.5	<0.001
Apo-A1 (g/L)		1.07 (1.00, 1.21)	1.21 (0.88, 1.51)	4.5	<0.001
HDL-C (mmol/L)		1.15 (1.00, 1.35)	1.28 (1.10, 1.45)	11.2	<0.001
LDL-C (mmol/L)		2.62 (2.12, 3.15)	3.00 (2.60, 3.40)	16.36	<0.001

GLU, glucose; TBIL, total bilirubin; DBIL, direct bilirubin; IBIL, indirect bilirubin; TC, total cholesterol; TG, triglycerides; APOA1, apolipoprotein A1; HDL-C, high-density lipoprotein cholesterol; LDL-C, low-density lipoprotein cholesterol.

The clinical characteristics of the participants detected mRNA are summarized in Supplementary Table S4. There were significant differences in age among SZ patients during the episode [(37.25 ± 13.39) years], patients in remission [(31.11 ± 7.82) years], and controls [(34.61 ± 10.87) years] (*F* = 5.01, *p* = 0.01). In addition, there were significant differences in GLU, TBIL, DBIL, IBIL, TG, and HDL-C among the three groups (all *p* < 0.01). There were no significant differences in sex, TC, Apo-A1, and LDL-C among these three groups (all *p* > 0.05).

### Association Analysis of Case–Control Study for SZ

The genotype and allele frequency distributions of rs5072 of *APOA1* and rs1268354 of *APOA4* between SZ cases and controls are summarized in Supplementary Table S5 in the Supplement. The allele frequency distributions of the two SNPs were consistent with the HWE (*p* > 0.1) in the controls.

Both rs5072 of *APOA1* and rs1268354 of *APOA4* were significantly associated with SZ even after adjustment for age and sex. The adjusted *ORs* (95% CIs) of the additive model for rs5072 (GG vs. GA vs. AA) and rs1268354 (CC vs. CT vs. TT) were 0.82 (0.75–0.90) and 1.12 (1.03–1.23), respectively, and *p*-values were 3.22 × 10^−5^ and 0.011. The associations of rs5072 with SZ still presented statistical significance even after Bonferroni correction (*p*-value×6).

### Sensitivity Analysis by Propensity Score Matching Model

We conducted a sensitivity analysis using a propensity score matching model to match SZ cases and controls by age and sex. There were no difference in average age and sex between SZ case and controls (*p* > 0.30). The serum levels of Glu, DBIL, TC, TG, ApoA1, HDL-C, and LDL-C were significantly lower in SZ cases than those of controls with all *p* values less than 0.01 (Supplementary Table S6).

Both rs5072 of *APOA1* and rs1268354 of *APOA4* were significantly associated with SZ even after adjustment for age and sex. The adjusted OR (95% CI) of the additive model for rs5072 was 0.84 (0.75–0.94) with *p*-values of 0.003. The association of rs5072 of *APOA1* and SZ still presented statistical significance even after Bonferroni correction (*p*-value×6). *OR* (95% CI) of the recessive model (TT vs. CT + CC) for rs1268354 was 1.31 (1.07–1.61) with a *p* value of 0.010. The OR of the subjects selected by the propensity score matching model was not different from that of the whole study population (less than 3%) (Supplementary Table S7).

### Stratification Analysis by Sex

Further stratified analysis by sex indicated that rs5072 was significantly associated with SZ both in men and women, and the association was homogeneous. rs1268354 of *APOA4* was significantly associated with SZ in women but not in men with *p*-value of heterogeneity test less than 0.05. In women, *OR* (95% CI) of the additive model for rs1268354 was 1.26 (1.11–1.45) with a *p*-value of 4.25 × 10^−4^, after adjustment for age (Table 2).

**TABLE 2:** Association analysis of *AOPA1* and *AOPA4* with schizophrenia in men and women.

Gene	SNP	Sex	Group	Genotype	Minor allele	OR (95% CI)^‡^, *p*-value^‡^					
				WT/HT/MT		Additive model		Dominant model		Recessive model	
APOA1	rs5072			GG/GA/AA	A		Heterogeneity test		Heterogeneity test		Heterogeneity test
		Men	Case	622/492/114		0.82 (0.72–0.94)	*p* = 1.00	0.80 (0.67–0.95)	*p* = 0.61	0.73 (0.55–0.96)	*p* = 0.47
			Control	626/569/160		*p* = 0.003		*p* = 0.01		*p* = 0.03	
		Women	Case	706/595/143		0.82 (0.72–0.94)		0.75 (0.62–0.89)		0.85 (0.64–1.14)	
			Control	368/390/97		*p* = 0.003		*p* = 0.001		*p* = 0.28	
APOA4	rs1268354			CC/CT/TT	T		Heterogeneity test		Heterogeneity test		Heterogeneity test
		Men	Case	445/571/205		1.00 (0.88–1.13)	*p* = 0.02	0.93 (0.78–1.11)	*p* = 0.03	1.14 (0.90–1.45)	*p* = 0.07
			Control	487/673/203		*p* = 0.99		*p* = 0.42		*p* = 0.28	
		Women	Case	514/677/253		1.26 (1.11–1.45)		1.25 (1.04–1.49)		1.60 (1.24–2.08)	
			Control	344/406/104		*p* = 4.25 × 10^−4^		*p* = 0.02		*p* = 3.35 × 10^−4^	

WT, wild type; HT, heterozygote; MT, mutant type. ‡: Adjusted for age.

### Comparing mRNA Levels Between SZ Cases and Controls

**FIGURE 1:**
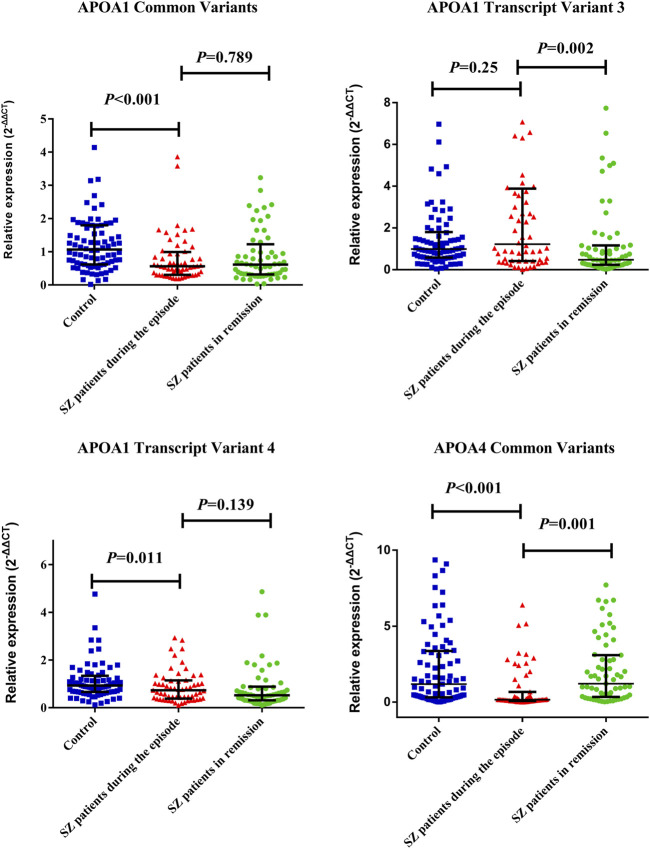
Distribution of APOA1 common transcript variants and transcript variant 4 of APOA1 among SZ patients during the episode, patients in remission, and controls. The dots represent individual data of mRNA levels (2^−ΔΔCT^). The upper line and lower line represent 75th percentage and 25th percentage, respectively, and the medium line represents the median. The medians (IRQs) of common variants and transcript variant 4 levels (2^-∆∆CT^) of *APOA1* mRNA in SZ during the episode (0.57 [0.31.0.99] (0.73 [0.38, 1.15]) were significantly lower than that of controls (1.07 [0.62.1.80] (0.93 [0.66.1.34]). Both the *p*-values were less than 0.05. SZ patients in remission showed a significantly decreased *APOA1* transcript variant 3 expression level (0.47 [0.24.1.16]) and increased *APOA4* mRNA expression level (1.35 [0.35.3.12]) than patients during the episode as above (*p* < 0.01).

The medians (IRQs) of common variants and transcript variant 4 levels (2^-∆∆CT^) of *APOA1* mRNA in SZ patients during the episode (0.57 [0.31.0.99]; 0.73 [0.38, 1.15]) were significantly lower than those of controls (1.07 [0.62.1.80]; 0.93 [0.66.1.34]); both the *p*-values were less than 0.01 (Figure 1). The median (IRQ) of common variants level of *APOA4* mRNA was significantly lower in SZ patients during the episode [0.16 (0.08.1.29)] than controls [1.34 (0.33.3.63)]; *p*-value was less than 0.001.

SZ patients in remission showed a significantly decreased *APOA1* transcript variant 3 expression level (0.47 [0.24.1.16]) and increased *APOA4* mRNA expression level (1.35 [0.35.3.12]) than patients during the episode as above (*p* < 0.01). The data are listed in Supplementary Table S8.

### Comparing mRNA Expression Levels Among Genotypes

The distribution of *APOA1* gene relative expression levels (2^-∆∆CT^) among the genotypes of rs5072 and rs1268354 is summarized in Supplementary Table S9 and Supplementary Table S10. The transcript variant 4 expression levels of *APOA1* mRNA significantly increased with the variations of rs5072 in SZ patients during the episode (*p*_trend_ = 0.017) but not in controls; the results are shown in Supplementary Table S9 in the Supplement.

### Partial Correlation Analysis of Serum apoA1, HDL-C, and mRNA Levels

Partial correlation analysis showed that apoA1 level was significantly correlated with HDL-C level in the controls (*r* = 0.89), SZ patients during the episode (*r* = 0.82), and patients in remission (*r* = 0.86) (all the *p*-values less than 0.001) after adjusting age and sex. However, mRNA levels of APOA4 showed no correlation with APOA1 in SZ cases and controls (Supplementary Table S11). In controls, *APOA1* transcript 3 was significantly correlated with apoA1 and HDL-C levels (*r* = −0.37, *p* = 0.001; *r* = −0.30, *p* = 0.006) and the correlations were found in women (*r* = −0.46, *p* < 0.001; *r* = −0.40, *p* = 0.006) but not in men (Supplementary Table S11), whereas the correlations were not observed in SZ cases.

However, no significant correlation of *APOA1* mRNA and *APOA4* mRNA was observed in SZ cases and controls (Supplementary Table S12).

### Comparing ApoA1 and HDL-C Levels Between SZ Cases and Controls

**FIGURE 2:**
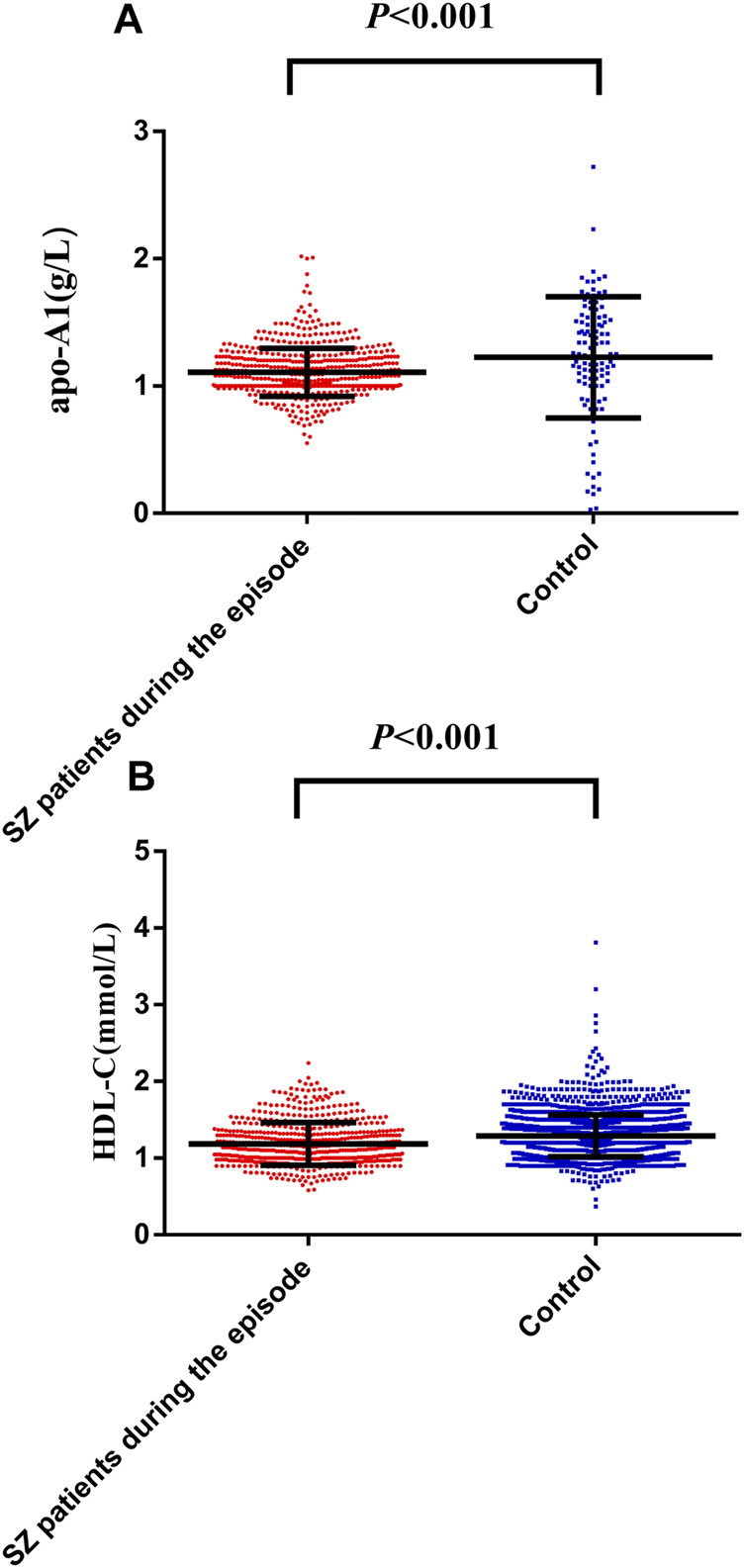
Distribution of apoA1 **(A)** and HDL-C **(B)** levels between SZ patients during the episode and controls. The dots represent individual data of apoA1 and HDL-C levels. The upper line and lower line represent 75th percentage and 25th percentage respectively, and the medium line represents the median. The serum apoA1 [median (IQR)] was significantly lower in SZ patients during the episode [1.07 (1.00.1.21) g/L] than controls [1.21 (0.88.1.51) g/L], *Z* = 4.04, *p* < 0.001 (Figure 2A). Similarly, HDL-C level in SZ during the episode [1.14 (0.99.1.34) mmol/L] was lower than controls [1.28 (1.10.1.45) mmol/L], *Z* = 9.02, *p* < 0.001 (Figure 2B).

The serum apoA1 [median (IQR)] was significantly lower in SZ patients during the episode [1.07 (1.00.1.21) g/L] than controls [1.21 (0.88.1.51) g/L], *Z* = 4.04, *p* < 0.001 (Figure 2A). Similarly, HDL-C level in SZ patients during the episode [1.14 (0.99.1.34) mmol/L] was lower than controls [1.28 (1.10.1.45) mmol/L], *Z* = 9.02, *p* < 0.001 (Figure 2B). All data are listed in Supplementary Table S12 in detail.

### Comparison of ApoA1 and HDL-C Levels Before and After Antipsychotic Treatment

**FIGURE 3:**
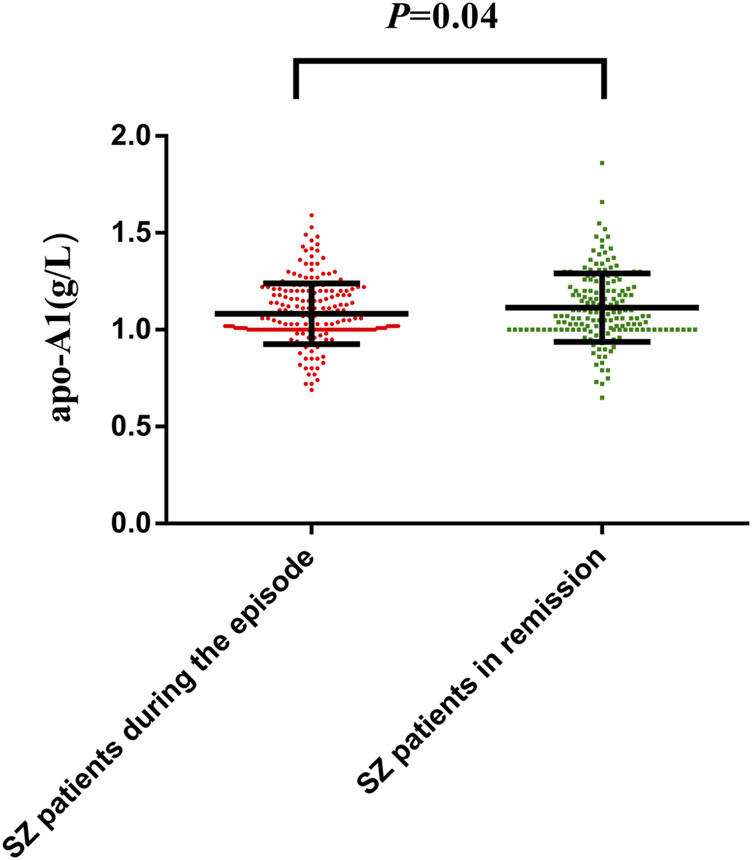
Distribution of apoA1 levels in SZ patients during the episode and patients in remission with antipsychotic treatment. The dots represent individual data of apoA1. The upper line and lower line represent 75th percentage and 25th percentage, respectively, and the medium line represents the median. Comparison of apoA1 and HDL-C levels before and after antipsychotic treatment was assessed by Wilcoxon test. The apoA1 levels of 209 SZ patients during the episode [1.03 (1.00.1.20) g/L] significantly increased after receiving an average 27.50 ± 9.90 days antipsychotic treatment [1.08 (1.00.1.22) g/L], *p*-value was 0.04 (Figure 3).

The apoA1 levels of 209 SZ patients during the episode [1.03 (1.00.1.20) g/L] significantly increased after receiving an average 27.50 ± 9.90 days antipsychotic treatment [1.08 (1.00.1.22) g/L]; *p*-value was 0.04 (Figure 3). However, the serum HDL-C level was not varied after treatment (*p* > 0.05). All the data are shown in Supplementary Table S13.

### Comparison of Serum ApoA1 and HDL-C Levels Among the Genotypes

In SZ patients during the episode, serum HDL-C levels [median (IRQ)] showed significant difference among the genotypes of rs5072 GG [1.10 (0.97, 1.33) mmol/L], GA [1.20 (1.00.1.35) mmol/L], and AA [1.10 (0.99.1.27) mmol/L], *p* < 0.05 (Supplementary Table S14). There was no significant difference in serum apoA1 level among the genotypes of rs5072 and rs1268354 (Supplementary Table S15) in SZ cases or controls.

## Discussion

In this study, we reported for the first time that variants at rs5072 of *APOA1* and rs1268354 of *APOA4* were significantly associated with SZ in a Chinese Han population. It is remarkable that the expression of *APOA1* mRNA was significantly down-regulated in peripheral blood leukocytes in the SZ patients during the episode.

*APOA1* gene encodes the apoA1 protein that belongs to the apolipoprotein A1/A4/E family (Wang et al., 2009). Rs5072 (G/A) is located in the intron region and is predicted to affect the binding of the transcription factor c-Myb (proximal promoter DNA-binding transcription activator activity), which regulates the expression of *APOA1* (http://alggen.lsi.upc.es/, last accessed September 1, 2018). The results of this study indicated that mRNA expression levels of *APOA1* transcript variant 4 significantly increased with the variations of rs5072 in SZ cases during the episode. Therefore, further functional research on the role of rs5072 and c-Myb in the regulation of mRNA expression would be warranted.

In this study, we observed lower serum apoA1 levels in SZ patients during the episode, compared with controls. This result is consistent with previous reports that apoA1 level decreased in the cerebrospinal fluid (CSF), liver, peripheral red blood cells, serum, and plasma in SZ patients (La et al., 2007; Huang et al., 2008). These findings suggest that the decreased apoA1 is involved in the pathology of SZ. It is well known that apoA1 is the primary core component required for normal HDL-C synthesis. The result in the present study indicated that apoA1 level is significantly correlated with HDL-C level. Moreover, we found that HDL-C levels were also lower in SZ patients during the episode. Antipsychotic agents can increase apoA1 levels as part of their therapeutic actions (Martins-De-Souza et al., 2010). In this study, the serum apoA1 levels of SZ patients during the episode significantly increased with the atypical antipsychotic treatment for a period of time, suggesting the potential adverse effect of antipsychotic treatment on the apoA1 levels. Since serum apoA1 varied with *APOA1* mRNA expression and clinic antipsychotic treatment, serum apoA1 would be suggested to detect and evaluate SZ prognosis besides HDL-C detection.

ApoA1 and HDL-C were mainly synthesized in the liver. The evidence of this study indicated that the *APOA1* mRNA expression in peripheral blood leukocytes has a population-based association with serum the apoA1 and HDL-C, suggesting a possible regulatory mechanism involved in apolipoprotein synthesis in the liver. On the other hand, serum lipid may be independent of the brain, but an unfavorable circulating lipid profile may cause hyperlipidemia and subtle changes in brain lipid metabolism (Salter, 1992). The exploration of the molecular mechanism of SZ supported the role of glutamate, *γ*-aminobutyric acid (GABA) and 5-hydroxytryptamine (5-HT) (Meyer and MacCabe, 2012; Owen et al., 2016). Thus, the relationship between dyslipidemia and dysfunction of neurotransmitters also received widespread attention (Salter, 1992; Terao et al., 1997). The impact of ApoA1 in the process of cholesterol efflux(Remaley et al., 2001; Zeng et al., 2004; Nofer et al., 2006; Ji et al., 2011) and phospholipid efflux (Remaley et al., 2001) may affect the dysfunction of 5-HT involved in the pathology of SZ.

The genetic ablation of *APOA4* may accelerate AD pathogenesis in an APP/PS1 transgenic mouse model (Cui et al., 2011). Csazar et al. reported that the *APOA4* codon 360 mutation (C > T) is associated with the increased risk of AD patients (Csaszar et al., 1997; Deng et al., 2015). This study found a significant association of rs1268354 C to T variation with SZ, particularly in women. However, no significant association of the levels of *APOA4* mRNA and the variations of rs1268354 was observed. rs1268354 does not have a linkage locus nearby *APOA1*, and the largest *r* ^2^ (with rs10750098) is 0.474. Thus, further study on epigenetics would be warranted to investigate the role of *APOA4* genetic variation in the pathogenesis of SZ.

This study has several potential limitations. First, after adjustment for age and sex, potential confounding effect remains to influence the association because of the differences in sex and age between SZ cases and controls. Nevertheless, the sensitivity analysis by propensity matching for age and sex validated the main significance (OR alterations less than 3%). Thus, we would accept the main findings of this study as well as the rationality of the primary study design for the SZ genetic association study. Second, the SZ patients during the episode with mRNA expression data were different from those in remission, so the selection bias is inevitable. Third, we failed to design an allele-specific primer for transcript 1 and transcript 2 of *APOA1* mRNA. Therefore, the possible effect of missing transcripts on SZ needs to be verified. Last, the effects of antipsychotic treatment and disease duration on mRNA expression warrant further investigation.

## Conclusion

In conclusion, the genetic variations of *APOA1* rs5072 and *APOA4* rs1268354 were significantly associated with SZ, and the expression levels of *APOA1* and *APOA4* mRNAs in peripheral blood leukocytes decreased in SZ patients during the episode while *APOA1* transcript variant 3 decreased and *APOA4* increased after antipsychotic treatment. These findings support the contribution of the genetic variations of *APOA1* and *APOA4* to the susceptibility to SZ with inhibiting mRNA expression in peripheral blood leukocytes. Further research on the role of apoA1 and apoA4 metabolism in molecular mechanism SZ is still warranted.

## Data Availability

The raw data supporting the conclusion of this article will be made available by the authors, without undue reservation.
