# Supplementary material for: The Variants at APOA1 and APOA4 Contribute to the Susceptibility of Schizophrenia With Inhibiting mRNA Expression in Peripheral Blood Leukocytes
Source: Front Mol Biosci. 2021 Dec 6;8:785445. doi: 10.3389/fmolb.2021.785445 (PMC8685515; doi:10.3389/fmolb.2021.785445)
Supplement: Supplementary file 1 [file DataSheet1.pdf]

Supplementary Information

The Variants at *APOA1 a*nd *APOA4* Contribute to the Susceptibility of Schizophrenia with Inhibiting mRNA expression in Peripheral Blood Leukocytes

Yao Fan[1,2†], Jun Gao[3†], Yinghui Li[4†], Xuefei Chen[5], Ting Zhang[2], Weiyan You[3], Chong Shen[2*], Yong Xue[5*]

[1] Department of Clinical Epidemiology, Geriatric Hospital of Nanjing Medical University, Jiangsu Province Geriatric Institute, Nanjing, China

[2] Department of Epidemiology, School of Public Health, Nanjing Medical University, Nanjing, China

[3] Department of Neurobiology, Nanjing Medical University, Nanjing, China.

[4] Department of Medical Psychology, Huai'an Third Hospital, Huai'an, China

[5] Department of Medical laboratory, Huai'an Third Hospital, Huai'an, China

†These authors have contributed equally to this work and share the first authorship.

[*]Correspondence: Chong Shen, sc100@126.com; Yong Xue, xueyong3@126.com

Methods

Genotyping PCR reactions and conditions

PCR reactions were performed in a 5μl reaction mixture including 10ng DNA, 2.4μl 2×TaqMan® Universal PCR Master Mix. Identification of Individual genotypes was performed by Sequence Detection System 2.1 software (95% auto caller confidence level). The PCR conditions were 50°C for 2 min, 95°C for 10 min, 95°C for 15s and then 60°C for 1 min, and forty-five cycles of real-time PCR were performed. The successful call rates of these four SNPs were over 99%.

Reverse transcription reactions and conditions

The reverse transcription reactions were performed in a 20 µl reaction mixture including 0.3 µg RNA, 2 µl 5'gDNA Eraser Buffer, 1 µl gDNA Eraser，4µl Nuclease-Free Water，4µl 5 x Primescript Buffer 2, 1µl Enzyme Mix1 and 1µl RT Primer Mix. The PCR conditions were 67 °C for 15min，85 °C for 5s.

RT-PCR reactions and conditions

According to the NCBI Nucleotide database, the human *APOA1* has 4 transcript variants, transcript variant 1 to 4, and accession numbers are NM_000039.2, NM_001318017.1, NM_001318018.1, NM_001318021.1, respectively. *APOA4* has only one transcript variant and the accession number is NM_000482.3. Besides failing to design an allele-specific primer of transcript variant 1 and transcript variant 2 of *APOA1*, the present study detecting the mRNA expression levels of co-sequences of and the transcript variant 3 and 4 as well as the transcript variant 1 of *APOA4*. The primers were designed using Primer Bank (https://pga.mgh.harvard.edu/primerbank/) or Primer Premier 3 (http://bioinfo.ut.ee/primer3-0.4.0/).

Real-time PCR (RT-PCR) reactions performed in a 10 µl reaction mixture including 2 µl cDNA, 5 µl HieffTM qPCR SYBR® GEEN Master Mix, 2.6µl Nuclease-Free water, 0.2 µl upstream primer, and 0.2 µl downstream primer. The RT-PCR conditions were 95°C for 5 min, 95°C for 10s, 60°C for 20s and 72°C for 20s with forty cycles. The melting curve includes 1 cycle with 95°C for 15s, 60°C for 1min, and 95°C for 15s. Three parallel samples were set up with a standard deviation less than 0.5, and the average value was calculated.

Table S1 Regions, alleles, MAF, predicted functions of SNPs

SNPs	Chromosome	Position	Regions	Allele[a]	MAF[b]	Regulome[c]
rs5072	11	116212793	Intron	G/A	0.363	TF binding + DNase peak
rs10750098	11	116210778	upstream transcript variant	T/G	-	-
rs12718462	11	116210929	upstream transcript variant	T/G	0.042	-
rs12718464	11	116212611	Intron	G/A	0.036	TF binding
rs2070665	11	116212894	Intron	G/A	0.363	TF binding
rs632153	11	116215449	Intron	G/T	0.06	TF binding
rs1268354	11	116195788	downstream gene variant	C/T	0.417	eQTL + TF binding / DNase peak

a Major/minor allele.
b Minor allele frequency (MAF) in Chinese Han Beijing (CHB) population.
c http://www.regulomedb.org.

Table S2 Sequences of Probes and Primers of rs5072 and rs1268354

SNP	Probes and Primers (5'-3')			
rs5072	Probe	FAM-CATTCCGGTTTCTC-MGB	Probe	HEX-CATTCCAGTTTCTCCA-MGB
	Forward primer	TGTGACCCTGCCTGGAGATC	reverse primer	CCGAGTCCTCACCTAATATCTGATG
rs1268354	Probe	TTTGAGACAAATGCTT	Probe	TTTGAGACAAACGCTTG
	Forward primer	AGGAAAGGGAGGTGGCTAGACT	reverse primer	TCGAAAACCTCACAAGGAAAGAG

Table S3 Primer sequences for mRNA

mRNA		Sequence
APOA1 transcript variantion3	Forward primer	TCCTGCTGCCTGCCCCGGTC
	reverse primer	CTCCCCGTCAGGAAGAGC
APOA1 transcript variantion4	Forward primer	CTTCCTGACGGAGCCCCTGG
	reverse primer	GCCTTCAAACTGGGACACAT
APOA1	Forward primer	CCCTGGGATCGAGTGAAGGA
	reverse primer	CTGGGACACATAGTCTCTGCC
APOA4	Forward primer	CCCAGCAACTCAATGCCCT
	reverse primer	CCTTCAGTTTCTCCGAGTCCT
GAPDH	Forward primer	ACACACTCAGTGAACA
	reverse primer	ATGGACTCATCTTTGCCTATTGC

Table S4 Characteristics of the participants detected mRNA levels.

Variables	Groups	SZ during the episode n=61	SZ in remission n=64	Control n=84	*F*/χ^2/*Z*	*P*
Age		37.25±13.39	31.11±7.82	34.61±10.87	5.014	0.007
Gender	Male	28	28	34	0.57	0.449
	Female	33	36	50		
Family history of SZ	Yes	7(6%)				
	No	118(94%)				
GLU (mmol/L)		4.50(3.90,5.40)	4.00(3.80,4.40)	4.20(4.10,4.50)	1.19	0.001
TBIL (umol/L)		12.39±5.95	9.66±5.24	13.39±5.38	3.85	<0.001
DBIL (umol/L)		3.50(2.40,5.40)	3.00(2.20,3.88)	5.70(4.40,6.90)	7.29	<0.001
IBIL (umol/L)		7.30(4.90,11.10)	5.75(3.78,7.58)	6.70(5.10,9.40)	1.23	0.008
TC (mmol/L)		4.19±0.79	4.03±0.86	3.95±0.81	1.04	0.243
TG (mmol/L)		1.04(0.75,2.01)	1.57(1.06,2.26)	0.92(0.77,1.46)	3.48	<0.001
Apo-A1 (g/L)		1.30±0.25	1.27±0.26	1.34±0.24	1.28	0.207
HDL-C (mmol/L)		1.05±0.20	1.02±0.22	1.12±0.18	2.69	0.011
LDL-C (mmol/L)		2.33±0.60	2.34±0.67	2.29±0.63	0.47	0.895

GLU, glucose; TBIL, total bilirubin; DBIL, direct bilirubin; IBIL, indirect bilirubin; TC, total cholesterol; TG, triglycerides; APOA1, apolipoprotein A1; HDL-C, high-density lipoprotein cholesterol; LDL-C, low-density lipoprotein cholesterol.

Table S5 Association analysis of SNPs of *AOPA1* and *AOPA4* with schizophrenia.

Gene	SNPs	Groups	Genotype	*OR (95%CI)* [a], *P* value [a]			Allele	
			WT/HT/MT	Additive model	Dominant model	Recessive model	Major/Minor	*OR(95%CI), P*[b]
APOA1	rs5072		GG/GA/AA				G/A	
		Case	1328/1087/257	0.82(0.75-0.90）	0.77(0.68-0.87）	0.79(0.65-0.96）	3743/1601	0.85(0.78-0.93)
		Control	994/959/257	P=3.22×10^{-5}*	P=3.64×10^{-5}*	P=0.021*	2947/1473	P=2×10^{-4}
APOA4	rs1268354		CC/CT/TT				C/T	
		Case	959/1248/458	1.12(1.03-1.23）	1.08(0.95-1.22）	1.34(1.13-1.59）	3166/2164	1.11(1.02-1.20)
		Control	831/1079/307	P=0.011*	P=0.254	P=0.001*	2741/1693	P=0.017

WT wild type, HT heterozygote, MT mutant type.

a Adjusted for age and gender.

b *P* value of χ^2 text for comparison of allele frequencies between case and control groups.

Table S6 Characteristics of participants in the subgroup of propensity matching analysis

Variable	Groups	Case (n=1387)	Control (n=1387)	$t/\chi^2/Z$	*P*
Age		41.83±9.76	41.40±10.06	27.86	0.30
Gender	Males	706 (50.9%)	706 (50.9%)		>0.99
	Females	681 (49.1%)	681 (49.1%)		
Family history of SZ	Yes	298 (21.5%)			
	No	1089(78.5%)			
GLU (mmol/L)		4.84 (4.3, 5.48)	5.3 (4.87, 5.77)	12.24	<0.001
TBIL (umol/L)		11.4 (8.11, 15.86)	11.14 (8.7, 15.17)	0.63	0.53
DBIL (umol/L)		4.28 (2.96, 5.97)	5 (3.4, 6.0)	3.40	<0.001
IBIL (umol/L)		7.07 (4.95, 10.41)	7.32 (5.09, 11.12)	0.68	0.49
TC (mmol/L)		4.11 (3.52, 4.82)	4.62 (4.0, 5.17)	12.20	<0.001
TG (mmol/L)		1.08 (0.77, 1.62)	1.28 (0.88, 2.04)	6.84	<0.001
Apo-A1 (g/L)		1.1 (1.0, 1.23)	1.21 (0.88, 1.51)	3.54	<0.001
HDL-C (mmol/L)		1.18 (1.0, 1.38)	1.23 (1.1, 1.4)	6.37	<0.001
LDL-C (mmol/L)		2.78 (2.24, 3.24)	3 (2.5, 3.39)	6.63	<0.001

Table S7 Sensitivity analysis of SNPs at *AOPA1* and *AOPA4* with schizophrenia by propensity matching

Gene	SNPs	Groups	Genotype	*OR* (95%*CI*) [a], *P* value [a]		
			WT/HT/MT	Additive Model	Dominant Model	Recessive Model
APOA1	rs5072		GG/GA/AA			
		Case	689/564/131	0.84 (0.75-0.94)	0.77 (0.67-0.90)	0.90 (0.70-1.15)
		Control	600/637/144	P=0.003	P=0.001	P=0.38
APOA4	rs1268354		CC/CT/TT			
		Case	509/632/238	1.11 (0.97-1.20)	1.01 (0.86-1.18)	1.31 (1.07-1.61)
		Control	513/681/190	P=0.16	P=0.93	P=0.01

WT wild type, HT heterozygote, MT mutant type.

a Adjusted for age and gender.

Table S8 Comparison of *APOA1* and *APOA4* mRNA expression levels between SZ cases and controls

mRNA	Groups	Healthy control ($2^{-\triangle\triangle CT}$)		SZ during the episode ($2^{-\triangle\triangle CT}$)		SZ in remission ($2^{-\triangle\triangle CT}$)		P^{a}	P^{b}	P^{c}
		n	Median (IQR)	n	Median (IQR)	n	Median (IQR)			
APOA1	Whole population	84	1.07(0.62,1.80)	60	0.57(0.31,0.99)	64	0.62(0.32,1.23)	<0.001	<0.001	0.79
	Men	34	0.63(0.47,1.62)	27	0.61(0.40,1.32)	28	0.55(0.27,1.33)	0.28	0.42	0.36
	Women	50	1.13(0.81,1.87)	33	0.53(0.30,0.85)	36	0.63(0.33,1.21)	<0.001	<0.001	0.22
APOA1 transcript variant 3	Whole population	82	0.99(0.59,1.80)	60	1.23(0.42,3.89)	62	0.47(0.24,1.16)	0.002	0.25	0.002
	Men	34	0.64(0.38,1.41)	27	1.29(0.44,4.16)	26	0.41(0.22,0.91)	0.004	0.058	0.002
	Women	48	1.20(0.88,1.98)	33	1.16(0.35,3.80)	36	0.69(0.29,1.74)	0.07	0.84	0.17
APOA1 transcript variant 4	Whole population	84	0.93(0.66,1.34)	61	0.73(0.38,1.15)	64	0.52(0.31,0.88)	<0.001	0.01	0.14
	Men	34	0.88(0.58,1.37)	28	0.77(0.36,1.32)	28	0.46(0.28,0.82)	0.02	0.28	0.11
	Women	50	0.96(0.74,1.33)	33	0.71(0.43,1.12)	36	0.59(0.34,1.23)	0.01	0.03	0.65
APOA4	Whole population	84	1.34(0.33,3.63)	61	0.16(0.08,1.29)	64	1.35(0.35,3.12)	<0.001	<0.001	<0.001
	Men	34	1.37(0.31,2.87)	28	0.18(0.11,2.34)	28	1.73(0.67,3.12)	0.007	0.02	0.004
	Women	50	1.28(0.33,3.95)	33	0.15(0.07,0.37)	36	0.93(0.25,3.78)	<0.001	<0.001	0.001

$\triangle\triangle CT_{case}=\triangle CT_{case}-\triangle CT_{control\ average\ value}$, $\triangle\triangle CT_{control}=\triangle CT_{control}-\triangle CT_{control\ average\ value}$, $\triangle CT=CT_{target\ gene}-CT_{housekeeper\ gene}$

a. *P* value for comparing among SZ during the episode, SZ in remission and controls

b. *P* value for comparing between SZ during the episode and controls

c. *P* value for comparing between SZ during the episode and SZ in remission

Table S9 Comparison of *APOA1* and *APOA4* mRNA levels among the genotypes of rs5072

Groups	mRNA($2^{-\triangle\triangle CT}$)	Groups	Genotypes				*P* for additive
			Num.	GG	GA	AA	
SZ during the episode	APOA1	Whole population	33/19/8	0.65(0.44,1.09)	0.45(0.26,0.67)	0.45(0.32,1.57)	0.102
		Men	18/6/3	0.72(0.38,1.19)	0.58(0.22,2)	-	0.871
		Women	15/13/5	0.59(0.53,1.03)	0.4(0.28,0.58)	0.37(0.24,1.55)	0.086
	APOA1 transcript variant 3	Whole population	33/19/8	0.9(0.37,3.71)	1.88(0.86,4.54)	0.83(0.61,6.12)	0.692
		Men	18/6/3	0.9(0.41,3.6)	4.15(1.07,8.26)	-	0.416
		Women	15/13/5	1.16(0.35,3.98)	1.55(0.51,3.46)	0.79(0.51,7.04)	0.98
	APOA1 transcript variant 4	Whole population	33/19/8	0.6(0.36,0.97)	0.77(0.34,1.38)	1.51(0.78,2.33)	0.02
		Men	18/6/3	0.45(0.31,0.98)	0.88(0.67,2.59)	-	0.015
		Women	15/13/5	0.73(0.47,0.97)	0.48(0.32,1.27)	1.02(0.58,9.45)	0.338
	APOA4	Whole population	33/19/8	0.2(0.09,2.83)	0.15(0.09,0.31)	0.12(0.05,0.16)	0.151
		Men	18/6/3	0.36(0.08,2.9)	0.15(0.1,0.36)	-	0.429
		Women	15/13/5	0.16(0.09,2.41)	0.15(0.07,0.33)	0.07(0.04,0.27)	0.403
SZ in remission	APOA1	Whole population	33/24/8	0.55(0.31,1.47)	0.66(0.33,1.27)	0.55(0.26,0.85)	0.918
		Men	13/10/5	0.34(0.22,1.81)	0.66(0.34,1.59)	0.55(0.24,0.82)	0.56
		Women	20/14/2	0.6(0.33,1.21)	0.68(0.29,1.07)	-	0.652
	APOA1 transcript variant 3	Whole population	33/24/8	0.45(0.19,1.45)	0.61(0.32,1.09)	0.46(0.22,1.01)	0.612
		Men	13/10/5	0.3(0.13,1)	0.61(0.31,0.93)	0.45(0.12,0.89)	0.331
		Women	20/14/2	0.71(0.19,1.65)	0.67(0.35,1.76)	-	0.737
	APOA1 transcript variant 4	Whole population	33/24/8	0.47(0.29,0.8)	0.69(0.48,1.2)	0.4(0.28,0.54)	0.132
		Men	13/10/5	0.32(0.21,0.48)	0.79(0.57,1.14)	0.4(0.23,0.5)	0.037
		Women	20/14/2	0.53(0.34,1.07)	0.64(0.28,1.39)	-	0.905
	APOA4	Whole population	33/24/8	1.48(0.31,2.49)	1.12(0.38,5.06)	2.2(0.21,4.43)	0.734

		Men	13/10/5	1.53(0.8,2.49)	2.51(0.97,4.79)	0.47(0.13,4.02)	0.304
		Women	20/14/2	0.93(0.2,2.6)	0.7(0.26,5.86)	-	0.462
Controls	APOA1	Whole population	40/37/7	1.1(0.62,1.85)	1.23(0.62,1.82)	0.75(0.37,0.85)	0.27
		Men	16/15/3	1.22(0.52,2.8)	0.62(0.38,1.56)	-	0.223
		Women	24/22/4	1.06(0.73,1.78)	1.26(0.91,1.9)	0.82(0.76,1.67)	0.36
	APOA1 transcript variant 3	Whole population	40/37/7	1.06(0.86,1.78)	0.96(0.55,2.12)	0.57(0.26,0.78)	0.122
		Men	16/15/3	0.92(0.47,2.73)	0.61(0.29,1.39)	-	0.171
		Women	24/22/4	1.27(0.92,1.78)	1.2(0.73,2.42)	0.77(0.38,7.12)	0.525
	APOA1 transcript variant 4	Whole population	40/37/7	1.01(0.77,1.22)	0.91(0.66,1.56)	0.62(0.46,0.71)	0.115
		Men	16/15/3	1.08(0.62,1.76)	0.86(0.55,1.36)	-	0.258
		Women	24/22/4	0.98(0.78,1.13)	0.99(0.75,2.4)	0.64(0.5,1.28)	0.264
	APOA4	Whole population	40/37/7	1.09(0.3,3.53)	1.59(0.31,4.19)	1.54(0.56,3.07)	0.739
		Men	16/15/3	1.14(0.26,3.33)	0.67(0.32,3.33)	-	0.899
		Women	24/22/4	1.06(0.33,3.7)	1.62(0.26,5.04)	1.82(0.48,7.01)	0.858

Table S10 Comparison of *APOA1* and *APOA4* mRNA levels among the genotypes of rs1268354

Groups	mRNA($2^{-\triangle\triangle CT}$)	Groups	Genotypes				*P* for additive effect
			Num.	CC	CT	TT	
SZ during the episode	APOA1	Whole population	22/28/10	0.61(0.48,0.89)	0.46(0.3,0.87)	0.71(0.37,1.6)	0.333
		Men	12/10/5	0.61(0.29,0.83)	0.79(0.4,1.34)	0.48(0.31,1.66)	0.858
		Women	10/18/5	0.63(0.53,1.07)	0.39(0.29,0.6)	0.94(0.33,1.55)	0.052
	APOA1 transcript variant 3	Whole population	22/28/10	0.69(0.35,4.02)	2.09(0.81,3.89)	0.88(0.52,3.56)	0.419
		Men	12/10/5	1.7(0.43,4.01)	1.72(0.77,7.99)	0.86(0.49,3.99)	0.657
		Women	10/18/5	0.44(0.11,4.56)	2.32(0.7,3.73)	1.04(0.46,3.88)	0.505
	APOA1 transcript variant 4	Whole population	22/28/10	0.64(0.35,0.97)	0.75(0.41,1.15)	1.16(0.4,2.05)	0.261
		Men	12/10/5	0.57(0.33,0.89)	0.62(0.39,2.27)	1.37(0.62,2.19)	0.24
		Women	10/18/5	0.64(0.4,1.01)	0.77(0.41,1.12)	0.48(0.38,9.45)	0.838
	APOA4	Whole population	22/28/10	0.2(0.09,2.8)	0.15(0.07,0.34)	0.14(0.12,0.25)	0.674
		Men	12/10/5	0.36(0.04,2.85)	0.23(0.15,2.12)	0.14(0.12,0.18)	0.533
		Women	10/18/5	0.19(0.09,3.41)	0.13(0.06,0.28)	0.16(0.11,1.48)	0.194
SZ in remission	APOA1	Whole population	24/29/11	0.65(0.46,1.56)	0.55(0.3,0.92)	0.47(0.26,1.65)	0.435
		Men	7/16/5	1.97(0.83,2.33)	0.47(0.3,0.77)	0.26(0.12,0.58)	0.024
		Women	17/13/6	0.6(0.39,0.82)	0.74(0.28,1.61)	1.5(0.43,2.26)	0.372
	APOA1 transcript variant 3	Whole population	24/29/11	0.75(0.22,3.29)	0.47(0.24,0.89)	0.45(0.24,1.01)	0.369
		Men	7/16/5	2.98(0.18,6.84)	0.38(0.22,0.88)	0.35(0.13,0.73)	0.445
		Women	17/13/6	0.75(0.22,2.2)	0.73(0.31,1.43)	0.45(0.24,2.5)	0.943
	APOA1 transcript variant 4	Whole population	24/29/11	0.53(0.32,1.57)	0.55(0.28,0.87)	0.4(0.19,1.16)	0.609
		Men	7/16/5	0.86(0.32,4.86)	0.46(0.26,0.73)	0.35(0.17,0.47)	0.076
		Women	17/13/6	0.51(0.32,0.8)	0.63(0.36,1.41)	0.91(0.32,3.37)	0.621
	APOA4	Whole population	24/29/11	1.35(0.27,2.8)	1(0.32,3.11)	2.02(0.47,5.09)	0.65

		Men	7/16/5	2.73(1,4.25)	1.63(0.92,3.12)	0.47(0.07,3.47)	0.29
		Women	17/13/6	0.92(0.18,2.39)	0.51(0.22,3.82)	3.65(1.65,5.86)	0.078
Controls	APOA1	Whole population	38/38/8	1.06(0.68,1.81)	1.09(0.61,1.78)	0.9(0.44,1.93)	0.97
		Men	14/15/5	1.25(0.51,1.6)	0.62(0.38,1.78)	0.64(0.36,2.92)	0.901
		Women	24/23/3	1.01(0.75,1.95)	1.21(0.82,1.8)		0.479
	APOA1 transcript variant 3	Whole population	38/38/8	0.99(0.56,1.63)	1.05(0.64,2.19)	0.67(0.31,1.86)	0.533
		Men	14/15/5	0.62(0.35,1.31)	0.74(0.41,2.83)	0.4(0.27,0.98)	0.366
		Women	24/23/3	1.2(0.89,1.87)	1.15(0.85,1.8)		0.846
	APOA1 transcript variant 4	Whole population	38/38/8	0.96(0.67,1.15)	1.03(0.66,1.54)	0.7(0.57,1.33)	0.606
		Men	14/15/5	0.97(0.55,1.35)	1.02(0.59,1.42)	0.69(0.51,8.03)	0.848
		Women	24/23/3	0.96(0.7,1.12)	1.04(0.77,1.84)		0.812
	APOA4	Whole population	38/38/8	1.81(0.31,4.98)	0.66(0.31,2.73)	1.57(0.99,3.08)	0.342
		Men	14/15/5	2.66(0.23,5.39)	0.67(0.36,1.74)	1.54(0.83,2.84)	0.475
		Women	24/23/3	1.38(0.38,4.73)	0.64(0.17,3.65)	1.59(0.81,.)	0.499

Table S11 Partial correlation between apoA1, HDL-C and mRNA levels

Protein	mRNA	Groups	Control		SZ during the episode		SZ in remission	
			r	***P***	***r***	***P***	***r***	***P***
ApoA1	APOA1	Whole	-0.035	0.758	0.029	0.840	0.094	0.476
		Men	-0.182	0.309	0.047	0.890	0.169	0.547
		Women	0.031	0.838	0.353	0.237	0.305	0.336
	APOA1 Transcript 3	Whole	-0.368	0.001	-0.133	0.353	0.218	0.094
		Men	-0.120	0.505	-0.199	0.558	0.467	0.080
		Women	-0.463	0.001	-0.494	0.086	-0.094	0.771
	APOA1 Transcript 4	Whole	0.067	0.553	0.233	0.100	0.033	0.802
		Men	0.193	0.281	0.550	0.079	0.283	0.307
		Women	0.002	0.989	-0.056	0.857	-0.066	0.840
	APOA4	Whole	-0.056	0.625	0.172	0.229	0.162	0.216
		Men	0.084	0.641	-0.002	0.996	-0.113	0.688
		Women	-0.140	0.348	0.461	0.113	0.610	0.035
HDL-C	APOA1	Whole	0.026	0.820	0.009	0.950	0.117	0.373
		Men	0.011	0.953	0.097	0.777	0.142	0.614
		Women	0.008	0.955	0.417	0.156	0.302	0.340
	APOA1 Transcript 3	Whole	-0.302	0.006	-0.093	0.515	0.175	0.181
		Men	-0.059	0.745	-0.023	0.946	0.207	0.459
		Women	-0.398	0.006	-0.403	0.172	-0.017	0.958
	APOA1 Transcript 4	Whole	0.164	0.146	0.075	0.602	0.003	0.983
		Men	0.355	0.043	0.658	0.028	0.157	0.577
		Women	0.005	0.739	-0.214	0.483	-0.061	0.851
	APOA4	Whole	0.071	0.532	0.044	0.758	-0.049	0.711
		Men	0.229	0.201	-0.085	0.804	-0.375	0.168
		Women	-0.023	0.876	0.263	0.384	0.554	0.062

Adjusted for age, gender, and antipsychotics in the whole study population and adjusted for age and antipsychotics in subgroup analysis.

Table S12 Partial correlation between mRNA of APOA1 and APOA4

		Control		SZ patients during the episode		SZ patients in remission	
**mRNA**	**Groups**	***r***	***P***	***r***	***P***	***r***	***P***
APOA1	Whole	0.02	0.88	0.17	0.21	0.17	0.2
	Men	0.02	0.92	0.28	0.17	0.07	0.74
	Women	-0.01	0.97	0.21	0.26	0.2	0.25
APOA1 Transcript 3	Whole	-0.04	0.7	-0.11	0.4	-0.05	0.71
	Men	-0.05	0.79	-0.11	0.59	-0.18	0.4
	Women	-0.05	0.76	-0.15	0.42	-0.03	0.88
APOA1 Transcript 4	Whole	0.02	0.86	-0.07	0.63	-0.02	0.87
	Men	0.02	0.93	-0.16	0.44	-0.13	0.55
	Women	0.03	0.84	-0.05	0.78	-0.01	0.97

Adjusted for age, gender, and antipsychotics in the whole study population and adjusted for age and antipsychotics in subgroup analysis.

Table S13 Analysis for comparison of apoA1(g/L) and HDL-C (mmol/L) levels between SZ during the episode and SZ in remission

Variables	Groups	SZ during the episode		Control		*Z*	*P*
		n	Median (IQR)	n	Median (IQR)		
ApoA1(g/L)	Whole Population	600	1.07(1.00,1.21)	326	1.21(0.88,1.51)	4.044	<0.001
	Men	262	1.05(1.00,1.20)	213	1.20(0.87,1.54)	2.878	0.004
	Women	338	1.09(1.00,1.21)	113	1.24(0.89,1.45)	2.986	0.003
HDL-C(mmol/L)	Whole Population	600	1.14(0.99,1.34)	2072	1.28(1.10,1.45)	9.015	<0.001
	Men	262	1.27(1.10,1.43)	1271	1.10(0.97,1.30)	7.695	<0.001
	Women	338	1.16(1.00,1.35)	801	1.28(1.10,1.45)	5.484	<0.001

Table S14 Analysis for comparison of apoA1(g/L) and HDL-C (mmol/L) levels between SZ during the episode and SZ in remission

**Variables**	**Groups**	**SZ during the episode**	**SZ in remission**	***Z***	***P***
ApoA1(g/L)	Whole Population (n=209)	1.03(1.00,1.20)	1.08(1.00,1.22)	2.012	0.044
	Men (n=115)	1.01(1.00,1.16)	1.03(0.95,1.22)	0.305	0.76
	Women (n=94)	1.09(1.00,1.22)	1.15(1.05,1.27)	3.235	0.001
HDL-C(mmol/L)	Whole Population(n=209)	1.14(0.99,1.34)	1.12(0.99,1.32)	1.089	0.276
	Men (n=115)	1.10(0.98,1.28)	1.03(0.95,1.22)	2.07	0.038
	Women (n=94)	1.19(1.00,1.44)	1.16(1.04,1.41)	0.655	0.512

Table S15 Comparison of serum apoA1 (g/L) and HDL-C (mmol/L) levels among the genotypes of rs5072

Variables	Groups	Genotypes	Control			SZ during the episode		
			n	Median (IQR)	*P* for additive effect	n	Median (IQR)	*P* for additive effect
ApoA1	Whole population	GG	139	1.21(0.89,1.52)	0.79	290	1.07(1.00,1.22)	0.75
		GA	162	1.22(0.84,1.50)		255	1.07(1.00,1.20)	
		AA	25	1.21(0.86,1.46)		55	1.08(1.00,1.25)	
	Men	GG	92	1.25(0.93,1.55)	0.25	133	1.06(1.00,1.21)	0.65
		GA	106	1.15(0.66,1.52)		108	1.05(1.00,1.19)	
		AA	15	1.26(0.88,1.48)		22	1.06(1.00,1.35)	
	Women	GG	47	1.17(0.78,1.43)	0.13	157	1.09(1.00,1.22)	0.82
		GA	56	1.29(1.02,1.48)		147	1.09(1.00,1.21)	
		AA	10	0.88(0.47,1.42)		33	1.08(1.00,1.23)	
HDL-C	Whole population	GG	927	1.30(1.10,1.45)	0.19	290	1.10(0.97,1.33)	0.05
		GA	900	1.25(1.10,1.44)		254	1.20(1.00,1.35)	
		AA	235	1.23(1.09,1.46)		55	1.10(0.99,1.27)	
	Men	GG	585	1.30(1.10,1.43)	0.74	133	1.07(0.965,1.3)	0.57
		GA	538	1.23(1.10,1.42)		107	1.11(0.99,1.31)	
		AA	141	1.26(1.09,1.50)		22	1.13(0.97,1.28)	
	Women	GG	342	1.30(1.10,1.48)	0.23	157	1.14(0.98,1.35)	0.07
		GA	362	1.28(1.10,1.45)		147	1.23(1.01,1.39)	
		AA	94	1.23(1.09,1.40)		33	1.09(0.96,1.24)	

Table S16 Comparison of serum apoA1 (g/L) and HDL-C (mmol/L) levels among the genotypes of rs1268354

			Control			SZ during the episode		
**Variables**	**Groups**	**Genotypes**	**n**	**Median (IQR)**	***P* for additive effect**	**n**	**Median (IQR)**	***P* for additive effect**
ApoA1	Whole population	CC	125	1.17(0.71,1.45)	0.194	224	1.05(1,1.2)	0.454
		CT	155	1.24(0.9,1.52)		266	1.075(1,1.21)	
		TT	46	1.23(0.9,1.54)		106	1.075(1,1.26)	
	Men	CC	74	1.14(0.72,1.46)	0.283	97	1.05(1,1.2)	0.870
		CT	100	1.23(0.89,1.56)		111	1.05(1,1.2)	
		TT	39	1.22(0.9,1.54)		52	1.02(1,1.22)	
	Women	CC	51	1.21(0.68,1.45)	0.528	127	1.05(1,1.19)	0.126
		CT	55	1.26(0.92,1.44)		155	1.1(1,1.21)	
		TT	7	1.37(0.89,1.69)		54	1.11(1,1.29)	
HDL-C	Whole population	CC	778	1.3(1.1,1.48)	0.337	224	1.1(0.97,1.3)	0.181
		CT	1005	1.26(1.1,1.43)		265	1.15(0.99,1.35)	
		TT	284	1.28(1.14,1.43)		106	1.165(1.00,1.43)	
	Men	CC	457	1.3(1.1,1.5)	0.121	97	1.1(0.96,1.28)	0.476
		CT	626	1.25(1.09,1.41)		110	1.089(0.99,1.34)	
		TT	186	1.23(1.11,1.4)		52	1.10(0.97,1.33)	
	Women	CC	321	1.27(1.07,1.46)	0.314	127	1.1(1,1.32)	0.137
		CT	379	1.28(1.1,1.45)		155	1.2(0.99,1.37)	
		TT	98	1.3(1.16,1.47)		54	1.24(1.02,1.48)	
